# Vestibular evoked myogenic potentials in the prognosis of sudden hearing loss ‒ a systematic review^☆^

**DOI:** 10.1016/j.bjorl.2019.10.001

**Published:** 2019-11-02

**Authors:** Nathalia de Paula Doyle Maia, Karen de Carvalho Lopes, Fernando Freitas Ganança

**Affiliations:** Universidade Federal de São Paulo (Unifesp), Departamento de Otorrinolaringologia e Cirurgia de Cabeça e Pescoço, Ambulatório de Otoneurologia, São Paulo, SP, Brazil

**Keywords:** Vestibular evoked myogenic potentials, Prognosis, Sudden hearing loss, Potencial evocado miogênico vestibular, Prognóstico, Surdez súbita

## Abstract

**Introduction:**

Sudden hearing loss is an otorhinolaryngological emergency that often leads to severe damage to the auditory and vestibular function. The vestibular evoked myogenic potential is a test that allows a noninvasive evaluation of the otolithic system function and vestibulospinal and vestibulo-ocular pathways.

**Objective:**

To evaluate the importance of vestibular evoked myogenic potential in determining the prognosis of patients with sudden hearing loss.

**Methods:**

A search for articles published up to December 2018 was performed in the PubMed, Cochrane, VHL and LILACS databases using MeSH descriptors. Retrospective and prospective articles were included containing cervical or ocular vestibular evoked myogenic potential in sudden hearing loss patients and information on associated vertigo and/or dizziness.

**Results:**

Sixteen of 62 initially selected articles met the inclusion criteria and were analyzed. Regarding the methodology of the evaluated studies, 8 studies were prospective, six were retrospective, one contained part of the data from a retrospective analysis and another part from a prospective analysis, and one study was cross-sectional. A total of 872 patients were evaluated (50.22% males and 49.77% females) with a mean age of 51.26 years. Four hundred and twenty-six patients (50.35%) had vertigo and/or dizziness associated with sudden hearing loss. The cervical vestibular evoked myogenic potential was performed in all studies, but only seven assessed the ocular vestibular evoked myogenic potential. The cervical vestibular evoked myogenic potential showed alterations in 38.65% of 846 evaluated ears, whereas ocular vestibular evoked myogenic potential showed alterations in 47.88% of 368 evaluated ears. The hearing recovery rate was analyzed by 8 articles, with 63.4% of 410 evaluated ears showing hearing recovery.

**Conclusions:**

The studies suggest that the assessment of the vestibular system using vestibular evoked myogenic potential seems to be important in the prognosis of sudden hearing loss. For better follow-up of patients with sudden hearing loss, the emphasis should not be limited to the cochlea, but also include the diagnosis and treatment of vestibular abnormalities, regardless of the presence of vertigo.

## Resumo

**Introdução:**

A surdez súbita é uma emergência otorrinolaringológica que frequentemente cursa com graves danos à função auditiva e vestibular. O potencial evocado miogênico vestibular é um exame que permite a avaliação não invasiva da função do sistema otolítico e das vias vestíbulo-espinhal e vestíbulo-ocular.

**Objetivo:**

Avaliar a importância do potencial evocado miogênico vestibular na determinação do prognóstico dos pacientes com surdez súbita.

**Método:**

Uma busca de trabalhos publicados até dezembro de 2018 foi realizada nos bancos de dados PubMed, Cochrane, BVS e Lilacs com descritores cadastrados no MeSH . Foram incluídos artigos retrospectivos e prospectivos que contivessem o potencial evocado miogênico vestibular cervical ou ocular em pacientes com surdez súbita e informações sobre vertigem e/ou tontura associados.

**Resultados:**

Dezesseis de 62 artigos selecionados inicialmente preencheram os critérios de inclusão e foram analisados. Quanto à metodologia dos trabalhos avaliados, 8 estudos foram prospectivos, seis retrospectivos, um continha parte dos dados oriunda de uma análise retrospectiva e outra parte de uma análise prospectiva e um estudo foi transversal. Foram avaliados 872 pacientes (50,22% do gênero masculino e 49,77% feminino) com média de 51,26 anos. Do total de pacientes, 426 (50,35%) apresentavam vertigem e/ou tontura associada à surdez súbita. O potencial evocado miogênico vestibular cervical foi realizado em todos os estudos, porém o potencial evocado miogênico vestibular ocular em apenas sete. O potencial evocado miogênico vestibular cervical apresentou alteração em 38,65% de 846 orelhas avaliadas, enquanto o potencial evocado miogênico vestibular ocular estava alterado em 47,88% das 368 orelhas avaliadas. A taxa de recuperação auditiva foi analisada por 8 artigos, 63,4% de 410 orelhas avaliadas apresentavam recuperação auditiva.

**Conclusões:**

Os estudos demonstram que a avaliação do sistema vestibular com o uso do potencial evocado miogênico vestibular parece ter importância no prognóstico da surdez súbita. Para melhor acompanhamento do paciente com surdez súbita a ênfase não deve se restringir à cóclea, mas também no diagnóstico e tratamento de alterações vestibulares, independentemente da presença de vertigem.

## Introduction

Sudden hearing loss (SHL) is an otorhinolaryngological emergency defined as a hearing loss of at least 30 dB, at three consecutive audiometric frequencies, of sudden onset within 72 h.^1^ In most cases, it manifests unilaterally in individuals in the fourth decade of life,^1^ with no gender preference.^2^ Its incidence in developed countries is estimated at 5–20 cases per 100,000 inhabitants/year.^1^

The pathophysiology of SHL is not yet fully established. Some associations with viral infections, vascular disorders, and autoimmune diseases have been reported in the literature.^3^ However, in most cases the etiology is still considered idiopathic.^1^

In addition to damage to the hearing function, SHL can lead to changes in vestibular function.^4^ It is believed that can be explained by the hypothesis of the extent of the disease due to anatomical proximity.^5^ Some studies show that hearing recovery seems to be better in patients with normal results in caloric testing and vestibular evoked myogenic potential (VEMP).^4^, ^6^ However, the association between hearing level and vestibular dysfunction in SHL patients remains inconclusive.^4^

Several medications have been investigated for the treatment of SHL. However, systemic corticosteroids have been recommended as the drug of choice.^7^ An alternative to systemic treatment is intratympanic therapy, which has a lower risk of systemic side effects, allowing medication to directly penetrate the cochlea and reach a high concentration, even when used at low doses. Intratympanic therapy is considered to be a second-line therapy and is recommended for cases where hearing recovery has not occurred after treatment with oral corticosteroids.^8^

Some factors related to prognosis have been described, such as age, presence of vertigo, severity, and audiometric pattern of hearing loss, VEMP, evoked auditory brainstem response (ABR), and otoacoustic emissions (OAE).^9^ Studies have observed that hyporeflexivity in caloric testing, the absence of V-wave in the ABR, the lack of response in VEMP, and absence of OAE are associated with a worse prognosis of SHL.^5^, ^10^, ^11^ However, there is still controversy regarding the results of these studies.

VEMP is a short-latency evoked potential that allows the noninvasive assessment of the otolithic system function and vestibulospinal and vestibulo-ocular pathways.^3^ It can be divided into cervical vestibular evoked myogenic potential (cVEMP) and ocular vestibular evoked myogenic potential (oVEMP). cVEMP assesses the saccule, inferior vestibular nerve, lateral vestibular nucleus, vestibulospinal tract, and sternocleidomastoid muscle. The oVEMP mainly reflects the function of the utricle and superior vestibular nerve.

In some studies, a greater number of patients with profound hearing loss have been found to have abnormal VEMP results.^4^ However, in some cases, no association was observed between VEMP alterations and hearing level.^4^ Therefore, the findings of the VEMP role in predicting the auditory prognosis of patients with SHL are still controversial.^12^

Finally, this review aims to evaluate the importance of VEMP in SHL, summarizing the available data on the alterations of this test in the prognosis of patients with this disease.

## Methods

### Data sources and search strategy

A systematic review on VEMP in SHL was performed using MeSH descriptors in the PubMed, Cochrane, VHL and LILACS databases for studies published until December 2018. After combining specific keywords (“sudden deafness, “sudden hearing loss”, “sudden sensorineural hearing loss, “VEMP”, “vestibular evoked myogenic potential”, “vestibular evoked myogenic potentials”), articles written in English, Portuguese and Spanish were selected manually. An additional bibliographic research was performed to provide specific information regarding VEMP and SHL.

### Eligibility criteria for study selection

The following eligibility criteria were used for inclusion in the analysis of this review: retrospective or prospective evaluation through cervical or ocular VEMP in patients with sudden hearing loss and information on associated vestibular symptoms. Case reports, book chapters, systematic reviews, and studies that did not provide sufficient information for the analysis in this project were excluded.

### Data extraction

The necessary information and data were extracted from the selected studies and quantified using a standardized procedure. The characteristics of each study were evaluated, such as year of publication, study design, age, gender, number of assessed patients, laterality, presence of vertigo and/or dizziness, moment of VEMP performance, abnormalities in cVEMP or oVEMP (the following were considered alterations: absent or asymmetric response and change in latency), auditory recovery and moment of the final audiometric assessment. The hearing recovery criteria was considered as any improvement from 10 decibels on the average of at least four frequencies of the initial tonal audiometry, regardless of the classification (total, partial or mild) used in the studies.

### Methodological quality assessment

The assessment of quality and risk of bias of the analyzed studies was performed using the Agency for health care research and quality (AHRQ) checklist.^13^ This list has 11 evaluation criteria, including source of information, inclusion and exclusion criteria, time period, consecutive patients, masking, quality assurance, explanation for exclusions, confounder control, incomplete data withdrawal, data collection and follow up. One item is scored as 1 if included in the article and 0 if it is not. A score of 8 or higher indicates a high-quality study (Table 1).

**Table 1:** Quality control of selected studies according to the Agency for Health Care Research and Quality (AHRQ) criteria.

Articles	Quality of the Article according to AHRQ											
	A	B	C	D	E	F	G	H	I	J	K	Score
Liu, J et al.	1	1	1	1	0	1	1	1	0	1	0	8
Chen, YH, Young, YH	1	1	1	1	0	1	1	1	0	0	1	8
Pogson et al.	1	1	1	1	1	1	1	1	0	1	1	10
Niu et al.	1	1	1	1	0	1	1	1	0	1	0	8
Lee et al.	1	1	1	1	1	1	0	1	0	1	0	8
Fujimoto et al.	1	1	1	0	1	1	0	1	0	1	0	7
Nagai et al.	1	1	1	1	0	1	0	1	0	1	1	8
You et al.	1	1	1	0	1	1	1	1	1	1	0	9
Oiticica, et al.	1	1	1	1	0	1	1	1	0	1	1	9
Ogawa et al.	1	1	0	1	0	1	1	1	0	1	1	8
Korres et al.	1	1	1	1	1	1	1	1	1	1	1	11
Stamatiou et al.	1	1	1	0	1	1	1	1	1	1	0	9
Hong et al.	1	1	1	0	0	1	1	1	1	1	1	9
Chen, CN, Young, YH	1	1	1	0	0	0	0	1	0	1	1	6
Iwasaki et al.	1	1	1	1	0	1	1	1	0	1	1	9
Wu, CC, Young, YH	1	1	1	1	0	0	0	1	0	1	1	8

A, source of information; B, inclusion and exclusion criteria; C: time period; D, consecutive patients; E, masking; F, quality assurance; G, explanation of exclusions; H, control of confounders; I, incomplete data withdrawal; J, data integrity; K, follow-up; 1, present; 0, not present or not clear.

## Results

**Figure 1:**
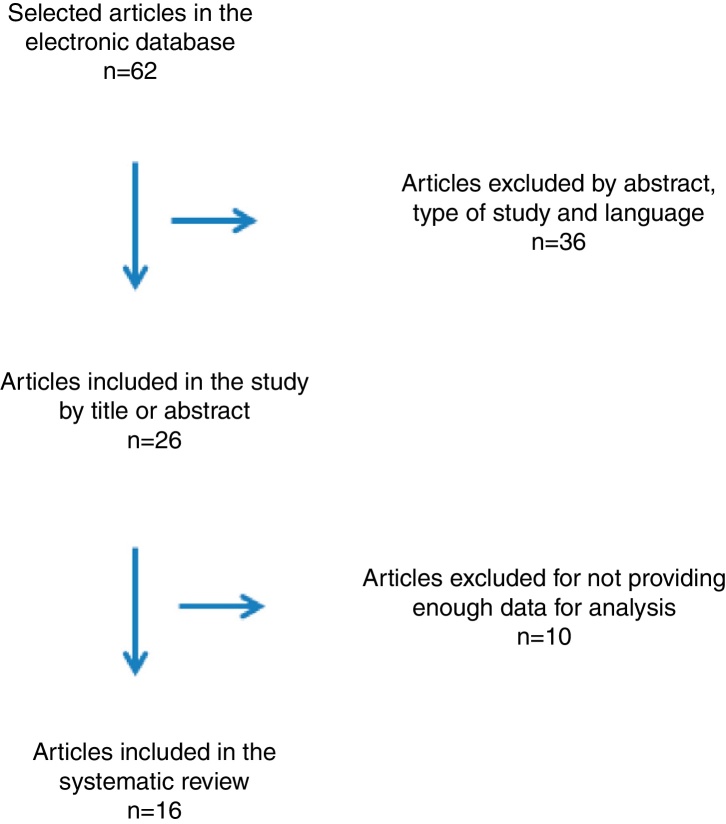
Flowchart of article selection for the systematic review.

According to the abovementioned criteria, 62 articles were initially selected. Of these, 25 were excluded by the abstract, 2 because they were systematic reviews, 1 because it was a book chapter and 8 articles because they were written in Chinese. Of the 26 remaining articles, 10 were excluded after reading the full article, as they did not provide sufficient information to verify the methodological quality required for inclusion in this study. Finally, 16 articles met the necessary criteria for the final review (Fig. 1).

Clinical data and complementary examinations of the 16 selected articles are listed in Table 2, in descending order of publication date, and the parameters used to perform VEMP (cervical and ocular) are listed in Table 3. Eight studies were prospective,^1^, ^12^, ^14^, ^15^, ^16^, ^17^, ^18^, ^19^ six were retrospective,^3^, ^4^, ^5^, ^6^, ^20^, ^21^ one study contained part of the data from a retrospective analysis and the other part from a prospective analysis (considered as retrospective/prospective),^22^ and one was a cross-sectional study.^23^ A total of 872 patients were evaluated, of which 50.22% were males and 49.77% females, with a mean age of 51.26 years. Four hundred and twenty-six patients (50.35%) had vertigo and/or dizziness associated with the SHL. cVEMP was performed in all articles, but oVEMP only in 7 of them.^1^, ^3^, ^4^, ^6^^,^^15^, ^21^, ^22^ Of 846 ears in which cVEMP was performed, 327 showed alterations in this exam (38,65%). The oVEMP showed abnormalities in 181 ears (47.88%), of the 378 evaluated ones. The auditory recovery rate was analyzed by only eight articles.^1^, ^5^, ^6^, ^12^^,^^14^, ^15^, ^18^, ^20^ Two hundred and sixty (63.4%) of 410 evaluated ears showed auditory recovery.

**Table 2:** Assessed characteristics of the selected studies.

Authors	Year	Study	Patients (M/F)	Laterality	Age	Vertigo and/or dizziness	Test performance	Altered cVEMP^a^	Altered oVEMP^b^	Auditory recovery – time^c^
Liu, J et al.	2017	R	35 (9/26)	U	41.9	21	‒	17 (48.5%)/35	22 (62.8%)/35	‒
Chen, YH, Young, YH	2016	P	5 (4/1)	B	45.6	‒	D	2 (100%)/2	4 (100%)/4	5 (50%) – 3
Pogson et al.	2016	R/P^d^	27 (17/10)	U	57.3	27	D^e^	9 (33.3%)/27	19 (70.3%)/27	‒
Niu et al.	2015	R	149 (72/77)	U	44.28	87	‒	73 (48.9%)/149	84 (56.3%)/149	‒
Lee et al.	2014	R	92 (55/37)	U	51.21	52	D	29 (31.5%)/92	‒	64 (69.5%) – 2
Fujimoto et al.	2014	R	25 (15/10)	U	63.6	25	‒	16 (64%)/25	10 (43%)/23	‒
Nagai et al.	2014	P	65 (35/30)	U	48.9	25	D	27 (41.5%)/65	6 (9.2%)/65	52 (80%) – 1
You et al.	2014	R	75 (42/33)	U	54	48	D	35 (47%)/75	36 (48%)/75	45 (60%) – 3
Oiticica, et al.	2013	C	21 (8/13)	U	52.5	‒	‒	5 (35.7%)/14	‒	‒
Ogawa et al.	2012	P	80 (43/37)	U	56.4	36	‒	24 (42.1%)/57	‒	47 (58.7%) – 1
Korres et al.	2011	P	104 (48/56)	U	52.5	36	D	30 (28.8%)/104	‒	‒
Stamatiou et al.	2009	P	86 (39/47)	U	51	31	D	26 (30.2%)/86	‒	‒
Hong et al.	2008	P	52 (22/30)	U	55.1	0	D	14 (26.9%)/52	‒	34 (65.3%) – 1
Chen, CN, Young, YH	2006	P	14 (7/7)	U	48	5	D	3 (21%)/21	‒	5 (35.7%) – 3
Iwasaki et al.	2005	R	22 (14/8)	U	54	22	D	17 (77%)/22	‒	8 (36.3%) – f
Wu, CC, Young, YH	2002	P	20 (8/12)	U	44	11	‒	0 (0%)/20	‒	‒

M, male; F, Female; P, Prospective; R, Retrospective; C, Cross-sectional; U, Unilateral; B, Bilateral; D, Tests performed at diagnosis or within 15 days; cVEMP, Cervical vestibular evoked myogenic potential; oVEMP, Ocular vestibular evoked myogenic potential;

a Number of ears with altered cVEMP (%)/total tested ears.

b Number of ears with altered oVEMP (%)/total tested ears.

c Ears that showed auditory recovery in relation to affected ears – moment (in months) of final audiometric evaluation.

d Until 2011, the study used data from retrospective analysis, when it became prospective.

e 2 patients underwent tests between 31–49 days after diagnosis and not on the day of diagnosis.

f Performed after several weeks (unspecified).

**Table 3:** Parameters used to perform VEMP (cervical and ocular) in the analyzed studies.

Authors	Year	cVEMP		oVEMP	
		Stimulus used	Alteration Criteria	Stimulus used	Alteration Criteria
Liu, J et al.	2017	Air conduction tone burst (500 Hz 100 dB nHL)	AR > 36%; reduced or absent amplitude; delayed response	Air conduction tone burst (500 Hz, 100 dB nHL)	AR > 40%; absent response
Chen, YH, Young, YH	2016	Bone conduction (500 Hz 144 dB force level)	‒	Bone conduction (500 Hz 144 dB force level)	‒
Pogson et al.	2016	Air conduction click (105 dB nHL 140 dB SPL)	AR > 39,6%	Bone conduction (147 dB force level)	AR > 39,9%
Niu et al.	2015	Air conduction tone burst (500 Hz 131 dB SPL)	Absent response	Air conduction tone burst (500 Hz 131 dB SPL)	Absent response
Lee et al.	2014	Air conduction click	Amplitude difference > 20% between ears; absent response	‒	‒
Fujimoto et al.	2014	Air conduction tone burst (500 Hz 95 dB nHL 135 SPL)	AR > 34%; absent response	Bone conduction tone burst (500 Hz 128 dB force level)	AR > 27,3%; absent response
Nagai et al.	2014	Air conduction click (105 dB nHL)	Ratio < 0,5	Bone conduction (500 Hz 115 dB force level)	AR > 49,7%; absent response
You et al.	2014	Bone conduction (500 Hz 128 dB force level)	AR > 33%; delayed response	Bone conduction (500 Hz 128 dB force level)	AR > 40%; absent response
Oiticica, et al.	2013	Air conduction tone burst (500 Hz 95 dB HL)	AR > 40%; absent response	‒	‒
Ogawa et al.	2012	Air conduction click (105 dB nHL)	Ratio < 0,5	‒	‒
Korres et al.	2011	Air conduction tone burst (500 Hz 95 dB HL)	Absent response	‒	‒
Stamatiou et al.	2009	Air conduction tone burst (500 Hz 95 dB HL)	Absent response	‒	‒
Hong et al.	2008	Air conduction click (95 dB nHL)	Late, Asymmetrical, or Absent Response	‒	‒
Chen, CN, Young, YH	2006	Air conduction tone burst (500 Hz 95 dB HL)	‒	‒	‒
Iwasaki et al.	2005	Air conduction click (95 dB nHL)	‒	‒	‒
Wu, CC, Young, YH	2002	Air conduction tone burst (500 Hz 95 dB HL)	Ratio > 0.33	‒	‒

−, not informed; Db, decibel; HL: hearing level; Hz, Hertz; AR, asymmetry ratio; Nhl, Normal Hearing Level; Ratio, ratio between the amplitude of the biphasic potential of the affected side and that of the healthy side; SPL, sound pressure level.

## Discussion

Sudden hearing loss is a relatively common event in otorhinolaryngology and has been extensively studied since its first description in the literature.^24^ However, to date its pathophysiology and the involved prognostic factors are not fully understood.^5^, ^25^ In addition to cochlear symptoms, SHL can also affect the vestibular system. However, it is important to note that the presence of dizziness is not mandatory, even with vestibular involvement. Several studies have shown that age, the presence of vertigo, the type of hearing loss on audiometry, the time between diagnosis and treatment, caloric testing and VEMP can be prognostic factors for this disease. However, many of these correlations are not yet fully established.^6^

Findings about prognosis and vestibular involvement in SHL are uncertain, which motivated this review. The VEMP is a complementary test, able to evaluate the function of otolithic organs. The cVEMP mainly evaluates the saccule and inferior vestibular nerve, while oVEMP evaluates the utricle and superior vestibular nerve. Therefore, we decided to analyze the importance of VEMP in the prognostic assessment of patients with SHL based on articles published in the literature.

The VEMP examination can be performed using click sound or tone burst stimuli in dB SPL (decibel sound pressure level) or dB HL (decibel hearing level).^26^ Because a high-intensity sound is required for auditory stimulation, there is concern about exposure to VEMP sound stimuli.^26^ The sound intensities should be limited to safe levels and the total energy delivered to the ear should be within acceptable limits.^26^ A 0.1 ms click of 139 dB SPL given at 5/sec and presented at each ear for up to 4.8 min, for instance, is within safe limits for sound exposure.^27^ Therefore, the way the exam is performed does not imply hearing damage.^26^, ^27^ It is also noteworthy that there was no hearing deterioration in patients after VEMP in all analyzed studies in this review.

Of the seven articles that simultaneously evaluated both exams (cVEMP and oVEMP), four showed a higher number of patients with changes in oVEMP than in the cVEMP^3^, ^4^, ^6^, ^22^; one article showed a similar number of changes in both exams^1^ and two had a higher number of patients with altered cVEMP compared to oVEMP.^15^, ^21^ Considering a larger number of patients with SHL and altered cVEMP in their studies, Fujimoto et al. and Nagai et al. suggested that the saccule could be more easily damaged than the utricle.^15^, ^21^ This could be related to the finding of microscopic temporal bone studies, which showed that loss of vestibular hair cells in SHL patients was more frequently observed in the saccule and less in the utricle and semicircular canals.^21^ On the other hand, the articles that showed a higher prevalence of patients with SHL and altered oVEMP suggested that the utricle could be more prone to damage than the saccule.^3^, ^4^, ^6^, ^22^ The authors state that this finding may be related to the fact that the bone canal is very narrow, making the superior vestibular nerve more susceptible to ischemic labyrinth changes or other complications when compared to the inferior vestibular nerve.^28^ Thus, it is still uncertain which otolithic organ is the one most often affected and, therefore, there is still insufficient data to state which VEMP (ocular or cervical) is more specific in determining prognosis.

The auditory recovery of SHL patients and associated factors was also analyzed in this review. Of the reviewed articles, six showed that altered VEMP in SHL patients was associated with poor hearing recovery.^1^, ^5^, ^6^, ^15^^,^^20^, ^21^ Hong et al. did not observe the association of altered cVEMP with the patients' poorer auditory prognosis.^12^ In contrast to the described articles, Wu, CC and Young, YH showed no alteration in cVEMP of the assessed SHL patients.^19^ Stamatiou et al. observed that the severity of vestibular lesion in SHL cases seems to increase with age, a fact attributed to the degeneration of vestibular structures that already occur in older individuals.^17^ Korres et al. suggested that more severe hearing loss and advanced age acted as independent negative predictive factors for auditory recovery, even with or without labyrinthine injury, assessed by cVEMP.^16^ The VEMP (cervical and ocular) was altered in 41.5% of the examined ears in the studies analyzed in this review. However, only eight studies analyzed the rate of auditory recovery, and the presence of altered VEMP was a worse prognostic factor in 6 of them. Therefore, the VEMP should be considered as a resource in the evaluation of patients with SHL.

Some limitations of the analyzed articles are not being multicentric or case-control or cohort studies; sample size; no data masking and lack of a prolonged follow-up. The investigation of sudden hearing loss with several tests, including vestibular evoked myogenic potential (cervical and ocular), Video Head Impulse Test (vHIT) and videonystagmography could better clarify the pathophysiology and extent (the structures affected in the inner ear) of the disease, in addition to being useful as a prognostic assessment tool for auditory recovery in patients with SHL.

## Conclusion

The studies suggest that the vestibular system assessment using VEMP seems to be important in the prognosis of sudden hearing loss. For better follow-up of patients with sudden hearing loss, the emphasis should not be limited to the cochlea, but also include the diagnosis and treatment of vestibular alterations, regardless of the presence of vertigo.

## Conflicts of interest

The authors declare no conflicts of interest.
